# Sequencing Directly from Clinical Specimens Reveals Genetic Variations in HCMV-Encoded Chemokine Receptor US28 That May Influence Antibody Levels and Interactions with Human Chemokines

**DOI:** 10.1128/Spectrum.00020-21

**Published:** 2021-10-27

**Authors:** Shelley Waters, Mark Agostino, Silvia Lee, Ibnu Ariyanto, Nina Kresoje, Shay Leary, Kylie Munyard, Silvana Gaudieri, Jessica Gaff, Ashley Irish, Anthony D. Keil, Patricia Price, Richard J. N. Allcock

**Affiliations:** a Curtin Medical School, Curtin Health Innovation Research Institute, Curtin University, Bentley, Western Australia, Australia; b Curtin Institute for Computation, Curtin University, Bentley, Western Australia, Australia; c Department of Microbiology, PathWest Laboratory Medicine WA, Nedlands, Western Australia, Australia; d Virology and Cancer Pathobiology Research Center, Faculty of Medicine, Universitas Indonesia, Jakarta, Indonesia; e School of Biomedical Sciences, University of Western Australia, Perth, Western Australia, Australia; f Institute for Immunology and Infectious Diseases, Murdoch University, Murdoch, Western Australia, Australia; g School of Human Sciences, University of Western Australia, Perth, Western Australia, Australia; h Department of Medicine, Division of Infectious Diseases, Vanderbilt University Medical Center, Nashville, Tennessee, USA; i Department of Nephrology, Fiona Stanley Hospital, Murdoch, Western Australia, Australia; j Department of Diagnostic Genomics, PathWest Laboratory Medicine WA, Nedlands, Western Australia, Australia; University of Arizona

**Keywords:** human cytomegalovirus, chemokine receptor, US28, renal transplant recipients, HIV patients, deep sequencing

## Abstract

Human cytomegalovirus (HCMV) is a beta-herpesvirus carried by ∼80% of the world’s population. Acute infections are asymptomatic in healthy individuals but generate diverse syndromes in neonates, solid organ transplant recipients, and HIV-infected individuals. The HCMV gene US28 encodes a homolog of a human chemokine receptor that is able to bind several chemokines and HIV gp120. Deep sequencing technologies were used to sequence US28 directly from 60 clinical samples from Indonesian HIV patients and Australian renal transplant recipients, healthy adults, and neonates. Molecular modeling approaches were used to predict whether nine nonsynonymous mutations in US28 may alter protein binding to a panel of six chemokines and two variants of HIV gp120. Ninety-two percent of samples contained more than one variant of HCMV, as defined by at least one nonsynonymous mutation. Carriage of these variants differed between neonates and adults, Australian and Indonesian samples, and saliva samples and blood leukocytes. Two nonsynonymous mutations (N170D and R267K) were associated with increased levels of immediate early protein 1 (IE-1) and glycoprotein B (gB) HCMV-reactive antibodies, suggesting a higher viral burden. Seven of the nine mutations were predicted to alter binding of at least one ligand. Overall, HCMV variants are common in all populations and have the potential to affect US28 interactions with human chemokines and/or gp120 and alter responses to the virus. The findings relied on deep sequencing technologies applied directly to clinical samples, so the variants exist *in vivo*.

**IMPORTANCE** Human cytomegalovirus (HCMV) is a common viral pathogen of solid organ transplant recipients, neonates, and HIV-infected individuals. HCMV encodes homologs of several host genes with the potential to influence viral persistence and/or pathogenesis. Here, we present deep sequencing of an HCMV chemokine receptor homolog, US28, acquired directly from clinical specimens. Carriage of these variants differed between patient groups and was associated with different levels of circulating HCMV-reactive antibodies. These features are consistent with a role for US28 in HCMV persistence and pathogenesis. This was supported by *in silico* analyses of the variant sequences demonstrating altered ligand-binding profiles. The data delineate a novel approach to understanding the pathogenesis of HCMV and may impact the development of an effective vaccine.

## INTRODUCTION

Human cytomegalovirus (HCMV) is carried by approximately 80% of the adult population globally (1). While acute infections are usually asymptomatic, seropositivity has been linked with accelerated cardiovascular disease (CVD) (2). Hence, flow-mediated dilation (FMD) of the peripheral vasculature (a measure of vascular endothelial dysfunction) can be considered a clinical footprint of HCMV (3). Acute HCMV disease generates diverse syndromes of end-organ disease in neonates, solid organ transplant recipients, and people living with HIV. We use the term “viral burden” as a metric for the latent and replicating HCMV present in an individual over time. The viral burden may be estimated from antibody levels, T-cell responses, populations of cells induced by HCMV infection, HCMV DNA (in blood or saliva), and viral microRNA (miRNA). The “HCMV footprint” describes the effects of viral burden on the immune system and on health outcomes. These concepts are reviewed in reference 3.

HCMV affects 20 to 60% of organ transplant recipients, precipitating graft rejection, symptomatic infections, and CVD (4). The level of risk is influenced by the type of organ transplanted, immunosuppressive medications, and prophylactic regimens. HCMV-seronegative renal transplant recipients (RTR) are at higher risk of primary infections with severe consequences, including graft loss and mortality (5).

Almost all individuals living with HIV are HCMV seropositive (6–8). HCMV retinitis is an AIDS-defining illness and is now rare (9), but HIV patients maintain higher levels of HCMV-reactive antibodies than healthy controls despite effective antiretroviral therapy (ART) (10). Higher antibody levels are associated with accelerated CVD and cerebrovascular disease (11).

The HCMV genome is approximately 235 kb in length (12) with 165 to 252 open reading frames (ORFs). However, only 45 ORFs are required for replication *in vitro* (13–16). Other ORFs are involved in immunomodulation, and many are homologs of host genes. This includes US28, an HCMV-encoded chemokine receptor expressed during the lytic and latent stages of infection (17, 18). US28 is most similar to C-X3-C motif chemokine receptor 1 (CX3CR1) (∼35% protein identity) and can bind the sole CX3CR1 ligand (19) CX3CL1. However, US28 can interact with 10 host chemokines, including C-C motif chemokine ligand 2 (CCL2) to CCL5 and CCL13 (20), and is an active coreceptor for HIV as it binds gp120 (21, 22). In RTR, US28 is expressed in vascular smooth muscle cells (VSMC) and tubular epithelial cells in kidney biopsy specimens collected during primary infections, reactivations, and latent infections. Furthermore, an HCMV variant with the US28 gene deleted has an impaired ability to spread through VSMC *in vitro* (23).

Studies addressing HCMV diversity through Sanger sequencing of PCR amplicons may miss multivariant infections, as the technique has limited capacity to detect variants present at frequencies less than 20%. In addition, several studies have sequenced HCMV that has been expanded *in vitro* and so may miss variants present *in vivo* (24, 25). Here, we describe nested PCR protocols with deep sequencing technologies applied to clinical samples. We present US28 gene sequences from RTR, HIV patients, healthy adults, and neonates. Patient US28 sequences were compared with a low-passage-number laboratory strain, Toledo, that was derived from the urine of a congenitally infected child (26).

## RESULTS

Targeted amplicon sequences targeting HCMV US28 were obtained from 60 clinical samples (blood, saliva, or urine) with a mean depth of 11,734. Twenty-eight samples were from Indonesian HIV patients (21 buffy coat and 7 saliva) collected after 0 to 3 months on ART, 21 were from Australian RTR (>2 years after transplant; 8 buffy coat and 13 saliva), 7 were from Australian healthy adults (2 buffy coat and 5 saliva), and 4 were from Australian neonates (urine). No adult donors had symptomatic infections.

### Most clinical samples contain more than one variant of HCMV.

**FIG 1:**
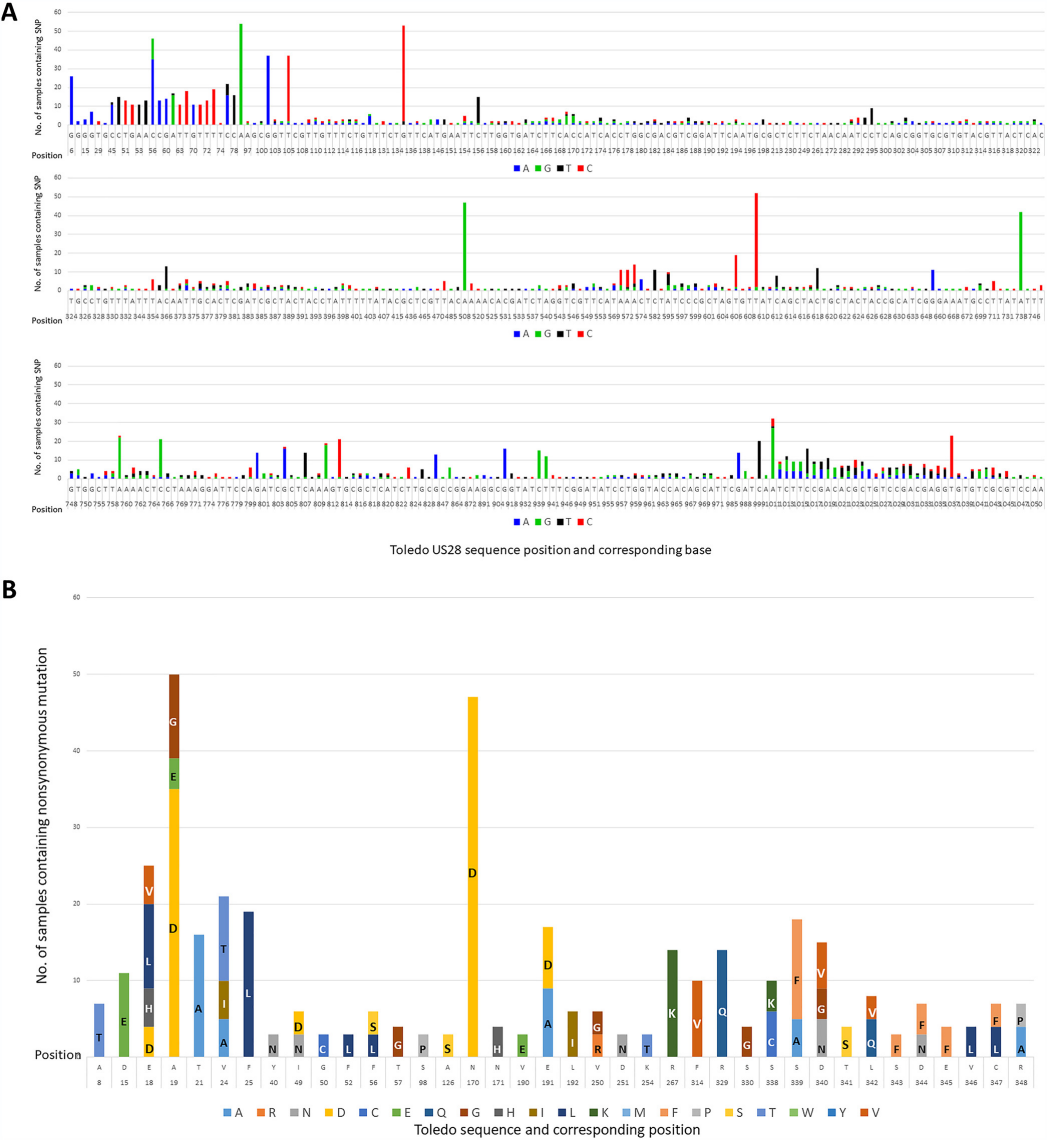
Summary of all nucleotide variations and nonsynonymous mutations identified in HCMV sequenced in 60 samples. (A) Nucleotide variations are displayed in reference to HCMV Toledo strain. Blue bars represent A, orange bars represent G, gray bars represent T, and yellow bars represent C. The height of the bars represents the number of samples the variation was present in. (B) Amino acid variations are displayed in reference to HCMV Toledo strain. Amino acids are represented by their one letter codes. Each variation presented was found in at least three samples. The height of the bars represents the number of samples carrying the variation.

**FIG 2:**
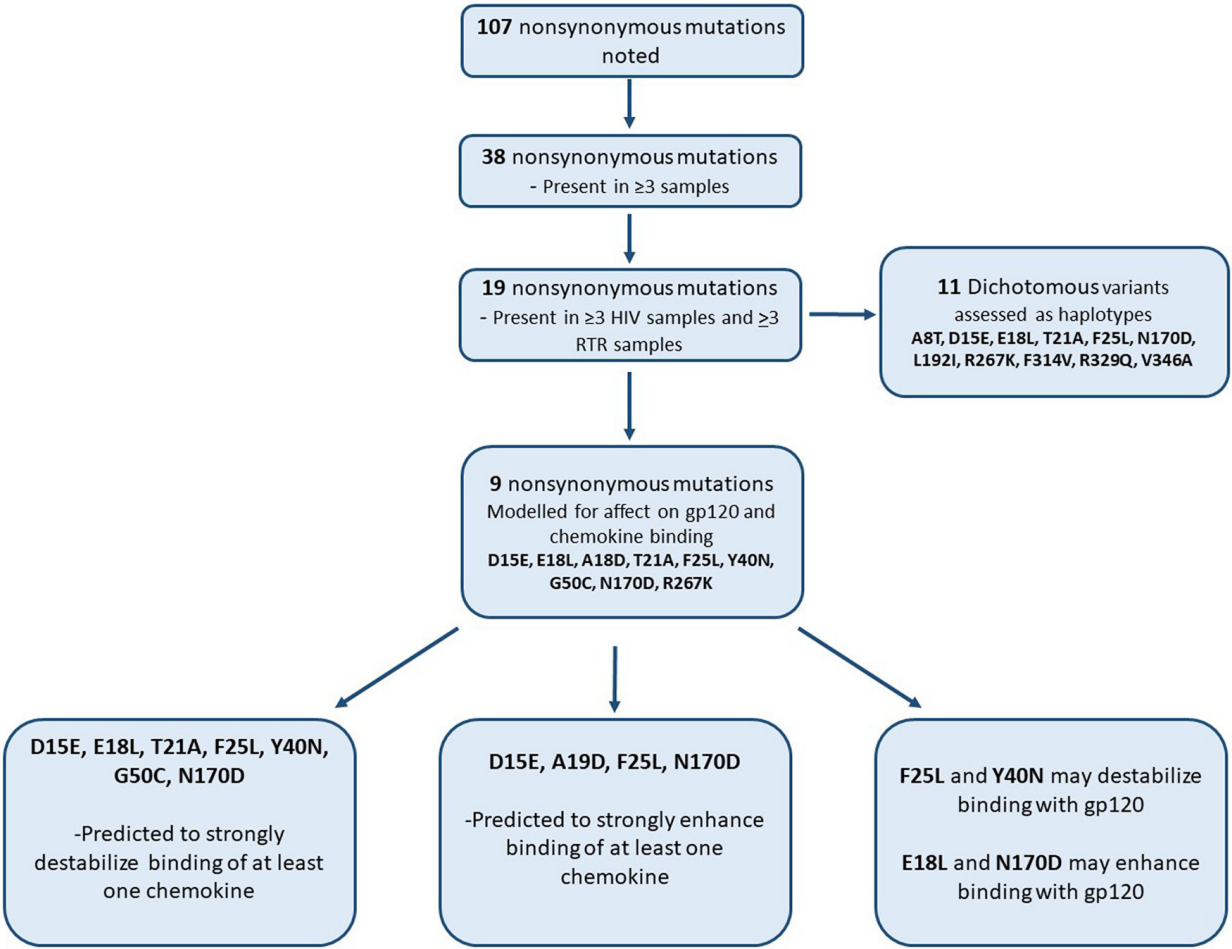
Flow diagram displaying the analysis of nonsynonymous variations in US28.

Compared with Toledo (GenBank no. GU937742.1), there were 430 sites of nucleotide variation (Figure 1A), of which 107 sites were nonsynonymous substitutions (Figure 1B). Thirty-eight substitutions were present in three or more samples, and 19 were present in six or more samples (Fig. 2). Of the 60 samples sequenced, 55 samples (∼92%) contained more than one variant of HCMV, based on the presence of nonsynonymous mutations in US28. None of the five samples with a single strain were 100% identical to the Toledo reference. The percentage of reads detecting each variant in every sample sequenced is presented in Table S1 in the supplemental material. Carriage of the variant sequences is discussed below.

### Several polymorphisms are group specific.

US28 sequences from neonates (*n* = 4) had 6 nonsynonymous variations, while sequences from adults (*n* = 56) had 46 nonsynonymous variations (Table 1). Forty of these were only seen in adults. US28 sequences from Australian donors (excluding neonates) had 42 nonsynonymous mutations, including 3 unique to Australian samples. HCMV sequences from Indonesians had 43 nonsynonymous mutations, including 3 unique to Indonesian samples.

**TABLE 1:** US28 protein variants distinct from Toledo were found in all groups

		Neonates	Adults	Australian	Indonesian	Buffy coat	Saliva
Residue position	Toledo reference	*n* = 4^*a*^	*n* = 56^*a*^	*n* = 28^*a*^	*n* = 28^*a*^	*n* = 31^*a*^	*n* = 25^*a*^
8^*b*^	A	A	A/**T**	A/T	A/T	A/T	A/T
15^*b*^	D	D	D/**E**	D/E	D/E	D/E	D/E
18^*b*^	E	E	E/**L**	E/L	E/L	E/L	E/L
19^*b*^	A	A/D	A/D/**G**	A/D/G	A/D/G	A/D/G	A/D/G
21^*b*^	T	T	T/**A**	T/A	T/A	T/A	T/A
24^*b*^	V	V	V/**A**/**T**	V/A/T	V/A/T	V/A/T	V/A/T
25^*b*^	F	F/L	F/L	F/L	F/L	F/L	F/L
40^*b*^	Y	Y	Y/N	Y/N	Y/N	Y/N	Y/N
49^*b*^	I	I	I/**D**	I	I/**D**	I/**D**	I
50^*b*^	G	G	G/**C**	G	G/**C**	G/**C**	G
52	F	F	F/**L**	F/L	F/L	F/L	F/L
56	F	F	F/**L**/**S**	F/**L**/S	F/S	F/L/S	F/L/S
57^*b*^	T	T	T/**G**	T	T/**G**	T/**G**	T
98	S	S	S/**P**	S/**P**	S	S/P	S/P
126	A	A	A/**S**	A/**S**	A	A/S	A/S
170^*b*^	N	N/D	N/D	N/D	N/D	N/D	N/D
171	N	N/H	N/H	N/H	N/H	N/H	N/H
190	V	V	V/**E**	V/E	V/E	V/**E**	V
191^*b*^	E	E	E/**A**/**D**	E/A/D	E/A/D	E/D	E/**A**/D
192^*b*^	L	L	L/**I**	L/I	L/I	L/**I**	L
250	V	V	V/**R**	V/R	V/R	V	V/**R**
251	D	D	D/**N**	D/N	D/N	D	D/**N**
254	K	K	K/**T**	K/T	K/T	K	K/**T**
267^*b*^	R	R/K	R/K	R/K	R/K	R/K	R/K
314^*b*^	F	F	F/**V**	F/V	F/V	F/V	F/V
329^*b*^	R	R	R/**Q**	R	R/**Q**	R/Q	R/Q
330	S	S	S/**G**	S/G	S/G	S/G	S/G
338	S	S	S/**C**/**K**	S/C/K	S/C/K	S/C/K	S/C/K
339^*b*^	S	S	S/**A**/**F**	S/A/F	S/A/F	S/A/F	S/A/F
340^*b*^	D	D	D/**N**/**G**/**V**	D/N/G/V	D/N/G/V	D/N/G/V	D/N/G/V
341	T	T	T/**S**	T/S	T/S	T/S	T/S
342	L	L	L/**Q**	L/Q	L/Q	L/Q	L/Q
343	S	S	S/F	S/F	S/F	S/**F**	S
344	D	D	D/**F**	D/F	D/F	D/F	D/F
345	E	E	E/**F**	E/F	E/F	E/F	E/F
346	V	V/A	V/A	V/A	V/A	V/A	V/A
347	C	C	C/**L**	C/L	C/L	C/L	C/L
348	R	R	R/**A**	R/A	R/A	R/A	R/A

a Nonsynonymous mutations are displayed in reference to Toledo. Changes unique to a group are in bold. All mutations reported were present in at least three samples.

b Mutations present in at least six samples and therefore studied further.

US28 sequences from buffy coat samples had 42 nonsynonymous variations, including 4 not found in urine or saliva. Sequences from saliva had 40 nonsynonymous variations, with 4 unique to saliva. Mutations A19D, F25L, N170D, N171H, R267K, and V346A were present in all groups and all sample types. N170D was the most frequent and was present in ∼90% (47/52) of samples. A19D was also abundant and was present in ∼63% (34/54) of samples.

### Amino acid haplotypes differ between samples from Australia and Indonesia.

Of the 38 nonsynonymous variations, 19 were present in at least six samples and were included in haplotyping analyses (Table 1). Only biallelic variations (11 positions) were included in amino acid haplotype models (Fig. 1). This identified 20 haplotypes (numbered US28-1 to US28-20), accounting for ∼82% of all genotypes (Table 2). D15E and E18L were always carried together (haplotypes 11, 17, 18, and 19) as were T21A and F25L in haplotypes 5, 6, 9, and 13, but not 16. US28-1 was more frequent in Australian than Indonesian samples (12/28 versus 1/28; *P* = 0.0009). US28-3 was only seen in Indonesian samples (5/28 versus 0/28; *P* = 0.05).

**TABLE 2:** Haplotype US28-1 is common in HCMV from Australian samples^*a*^

Toledo	A	D	E	T	F	N	L	R	F	R	V			
Variant	T	E	L	A	L	D	I	K	V	Q	A	Indo^*b*^	Aus^*c*^	*P* value^*d*^
Position	8	15	18	21	25	170	192	267	314	329	346	(*n* = 28)	(*n* = 28)	
US28-1	A	D	E	T	F	D	L	R	F	R	V	1	12	**0.0009**
US28-2	A	D	E	T	F	N	L	R	F	R	V	4	10	0.12
US28-3	A	D	E	T	F	D	L	R	F	Q	V	5	0	**0.05**
US28-4	A	D	E	T	F	D	L	R	F	R	A	8	3	0.18
US28-5	A	D	E	A	L	N	L	R	F	R	A	3	3	0.99
US28-6	A	D	E	A	L	N	L	R	F	R	V	1	4	0.35
US28-7	A	D	E	T	F	D	L	R	F	Q	A	4	0	0.11
US28-8	A	D	E	T	F	N	L	K	V	R	A	2	2	0.99
US28-9	A	D	E	A	L	N	L	R	F	Q	V	2	0	0.49
US28-10	A	D	E	T	F	N	L	R	F	Q	V	2	0	0.49
US28-11	T	E	L	T	F	D	L	R	F	R	V	3	1	0.61
US28-12	A	D	E	T	F	D	L	K	V	R	A	1	0	0.99
US28-13	A	D	E	A	L	D	L	K	V	R	A	0	3	0.24
US28-14	A	D	E	T	F	N	L	R	F	R	A	0	1	0.99
US28-15	A	D	E	T	F	N	L	K	F	R	A	0	1	0.99
US28-16	A	D	E	T	L	N	L	K	F	R	A	1	0	0.99
US28-17	T	E	L	T	F	D	L	R	F	Q	V	2	0	0.49
US28-18	T	E	L	T	F	N	L	R	F	Q	V	1	0	0.99
US28-19	A	E	L	T	F	D	L	R	V	R	V	0	3	0.34
US28-20	A	D	E	T	F	D	L	K	F	R	A	1	1	0.99

a Gray shading represents variation in comparison to Toledo reference.

b Indo, Indonesian samples.

c Aus, Australian samples.

d Fisher’s exact test comparing Australian and Indonesian adult samples; bold indicates that statistical significance was reached.

### Several variants are predicted to destabilize interactions between US28 and human chemokines.

**FIG 3:**
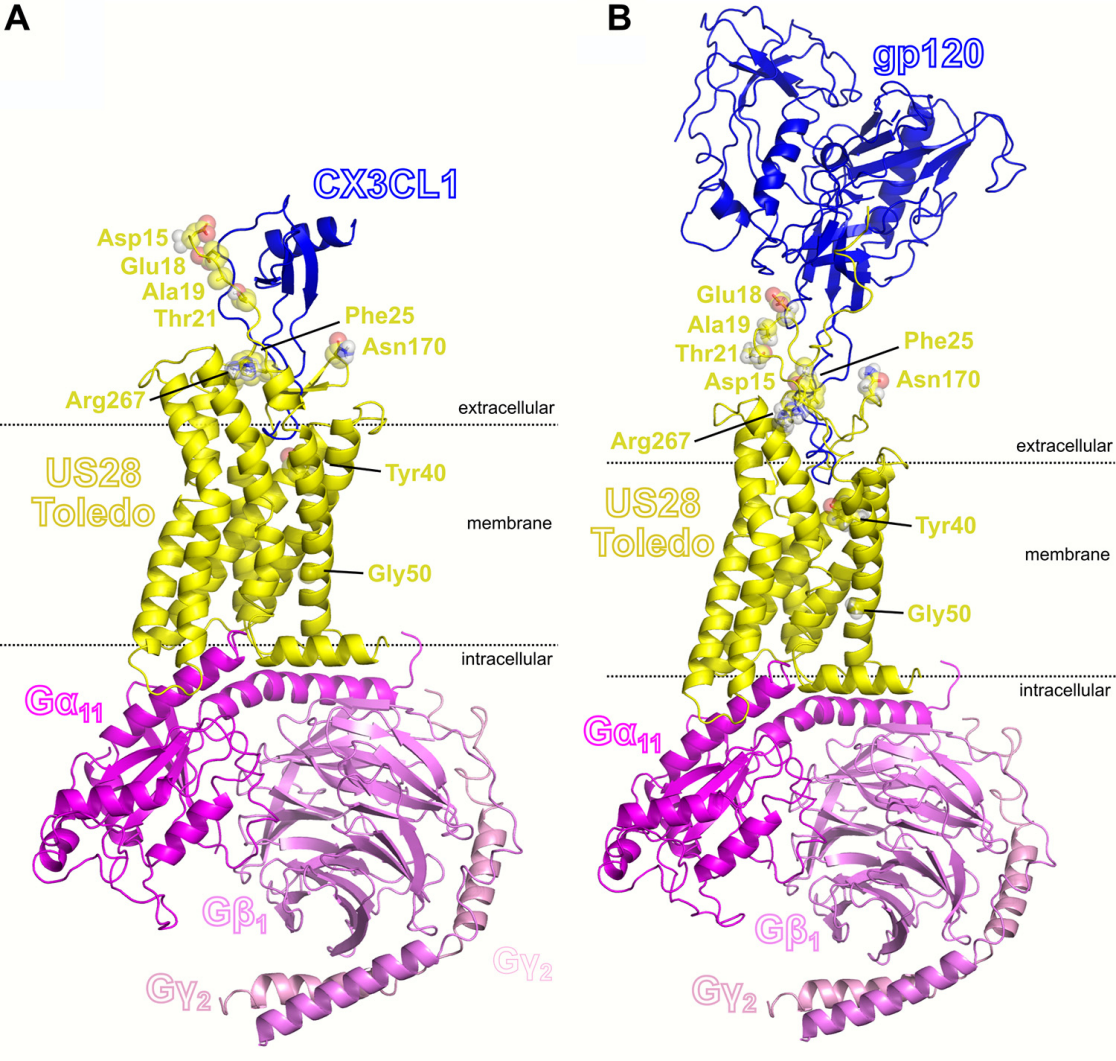
Model of Toledo US28-G protein complex binding with CX3CL1 (A) or gp120 (B). Residues of US28 urther investigated are shown as sticks with open spheres. G protein subunits are indicated by Gα_11_, Gβ_1_, and Gγ_2_.

Nine nonsynonymous variants were selected for modeling to predict interactions between US28 and human chemokines (CX3CL1, CCL2, CCL3, CCL4, CCL5, and CCL13) and HIV gp120 from Australia and Indonesia. The orientation is provided using CX3CL1 and gp120 in Fig. 3. Variants for further examination were selected as those within the amino acid range of US28 present in the modeled complexes with chemokines (residues 14 to 310).

Nine nonsynonymous mutations were selected based on differences in amino acid properties. Binding energies following mutation were calculated for the prepared complex structures using the Schrodinger Biologics Suite 2018-3 (see Materials and Methods). The results are summarized in Table 3 with variants arranged by their position in the protein sequence, and, in Fig. 2, D15E was predicted to destabilize binding of CCL3 and CCL13 and strengthen binding with CX3CL1, CCL2, and CCL4. E18L was predicted to destabilize binding of CX3CL1 and CCL3. A19D was predicted to enhance binding of CCL5, CCL2, and CCL13. T21A was predicted to destabilize binding of all chemokines to different degrees. F25L was predicted to weakly destabilize binding of all chemokines except CCL13. Y40N was predicted to strongly destabilize binding of all chemokines, with a ΔΔ*G* of greater than 10 kcal/mol for CX3CL1, CCL3, and CCL4. G50C (the only candidate distant from the chemokine-binding site of US28) was not predicted to destabilize binding of any chemokine except CCL4. Our models predicted that N170D would bind more strongly to CCL13 and more weakly to CCL2. R267K was predicted to have a minimal effect on binding of all chemokines.

**TABLE 3:** Predicted change in binding energy relative to Toledo (ΔΔ*G*, in kcal/mol) for interactions between clinical variants of US28 and chemokines or HIV gp120

Variant	CX3CL1 ΔΔ*G*	CCL2 ΔΔ*G*	CCL3 ΔΔ*G*	CCL4 ΔΔ*G*	CCL5 ΔΔ*G*	CCL13 ΔΔ*G*	gp120 Indo^*a*^ ΔΔ*G*	gp120 Aus^*b*^ ΔΔ*G*
D15E	−2.8	−4.3	+7.1	−2.6	−1.3	+4.0	+0.5	+1.3
E18L	+7.4	+1.6	+9.7	+0.7	+1.9	−0.8	−4.6	−3.9
D15E, E18L^*c*^	+4.0	−1.4	+15.0	−3.5	−0.7	+3.2	−5.3	−3.7
A19D	−1.2	−4.6	+0.2	+0.2	−6.6	−2.6	−1.0	−1.3
T21A	+2.4	+1.8	+7.5	+2.2	+2.8	+1.9	−0.3	+0.1
F25L	+1.6	+2.4	+1.5	+0.5	+0.4	−2.4	+7.1	+1.2
T21A, F25L^*c*^	+4.1	+4.3	+8.7	+2.3	+3.1	−0.6	+6.5	+1.5
Y40N	+14.5	+9.0	+10.6	+17.4	+9.6	+2.2	+2.9	+9.7
G50C	−0.0	−0.0	−0.0	+8.0	+0.8	−0.0	−0.0	+0.0
N170D	−0.4	+4.5	+2.2	+2.1	−0.1	−4.4	−1.8	−2.4
R267K	+0.9	+0.9	+0.5	+0.6	+0.2	+0.4	−0.3	+0.3

a Indonesian HIV isolate.

b Australian HIV isolate.

c Double mutations tested as alleles were universally coinherited.

Four out of nine variations were predicted to impact binding to gp120. F25L and Y40N could destabilize gp120 binding, while E18L and N170D could enhance gp120 binding. Furthermore, the models predicted that F25L could destabilize binding of gp120 sequenced from an Indonesian patient while having less effect on binding of the Australian gp120 sequence. Conversely, Y40N could substantially destabilize binding to the Australian gp120 with a smaller effect on binding to the Indonesian gp120 strain. N170D may enhance binding of Australian gp120 and moderately enhance binding of Indonesian gp120 (Table 3).

D15E, E18L and T21A, F25L were also examined pairwise because the variant alleles were coinherited (see Table 2). The D15E, E18L double mutant favors binding to gp120 and CCL4 but not other chemokines, while T21A, F25L is more likely to inhibit binding to chemokines or gp120.

In contrast, G protein binding was not typically predicted to be affected in any of the US28 variants regardless of the complex being examined. This included G50C, which is closest to the interface with the G protein. Exceptions were the CCL4-G50C and CCL5-G50C complexes (Table S2).

### US28 variations associate with levels of HCMV-reactive antibody.

The nine variations assessed by modeling were also assessed for correlations with levels of HCMV-reactive antibodies in plasma. Variants R267K and N170D associated with antibody levels and are presented here.

**FIG 4:**
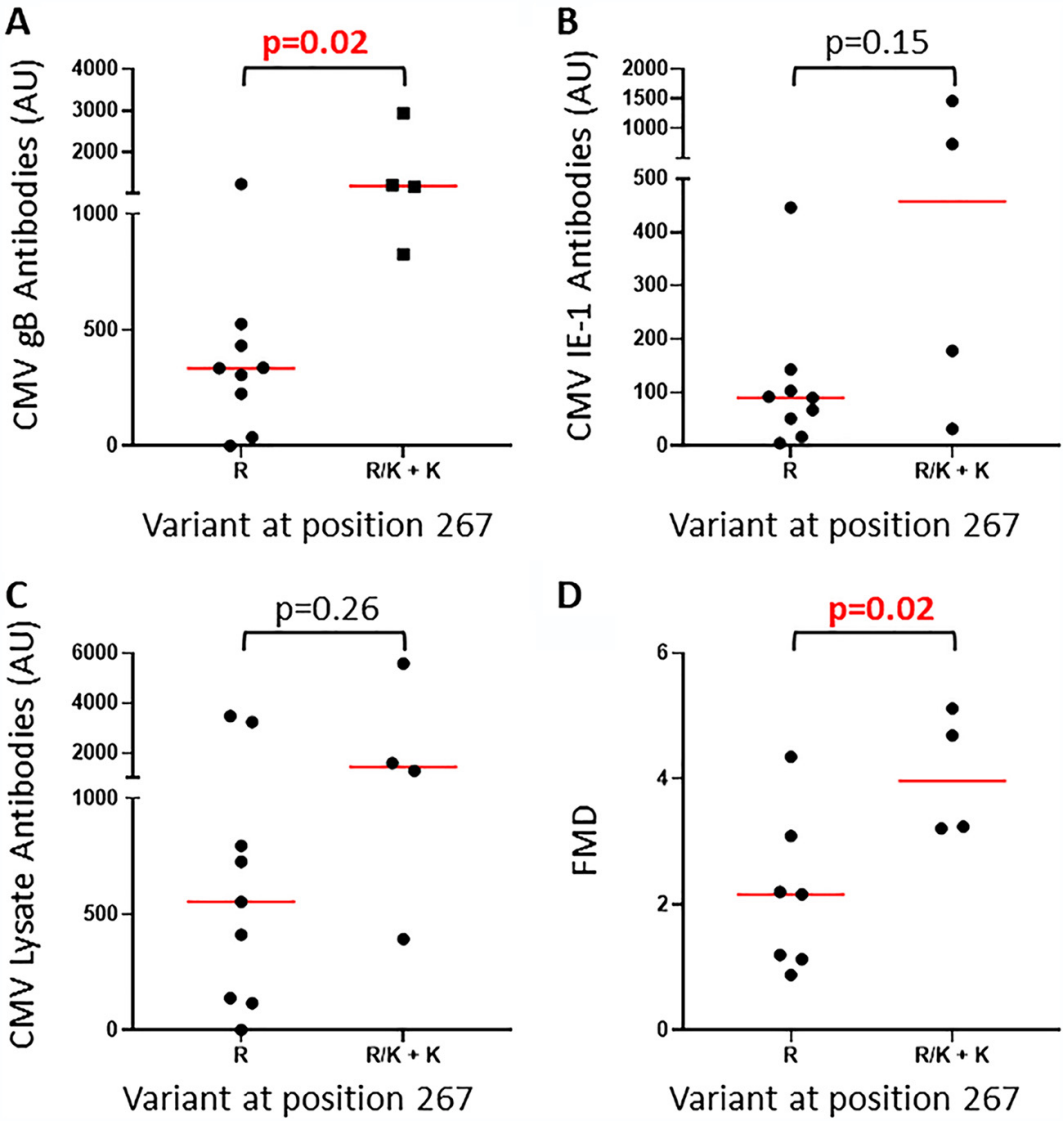
RTR carrying HCMV with the US28 R267K variant have higher HCMV-reactive antibody levels. (A to C) Comparison of HCMV gB (A), IE-1 (B), and lysate-reactive antibody (C) levels between RTR carrying HCMV with only R at position 267 and those carrying R/K or only K. (D) Comparison of flow-mediated dilation (FMD) between RTR carrying HCMV with only R at position 267 and those carrying R/K or only K.

HCMV encoding K at position 267 of US28 was present in all cohorts (14/60 samples), including 5/15 samples from RTR. This was sufficient to assess associations with measures of the burden and clinical footprint of HCMV in RTR. RTR carrying the R267K variant had higher levels of HCMV glycoprotein B (gB)-reactive antibodies (*P* = 0.02) (Fig. 4A) and with similar trends for immediate early protein 1 (IE-1) and CMV lysate-reactive antibodies (Fig. 4B and C). Furthermore, RTR carrying the R267K variant had higher FMD scores, marking superior cardiovascular health (*P* = 0.02) (Fig. 4D). All RTR described here had HCMV DNA detectable with a standard clinical assay. Levels of HCMV DNA were not affected by R267K (data not shown), perhaps because they were strongly time dependent or because CMV replicates in tissue cells.

**FIG 5:**
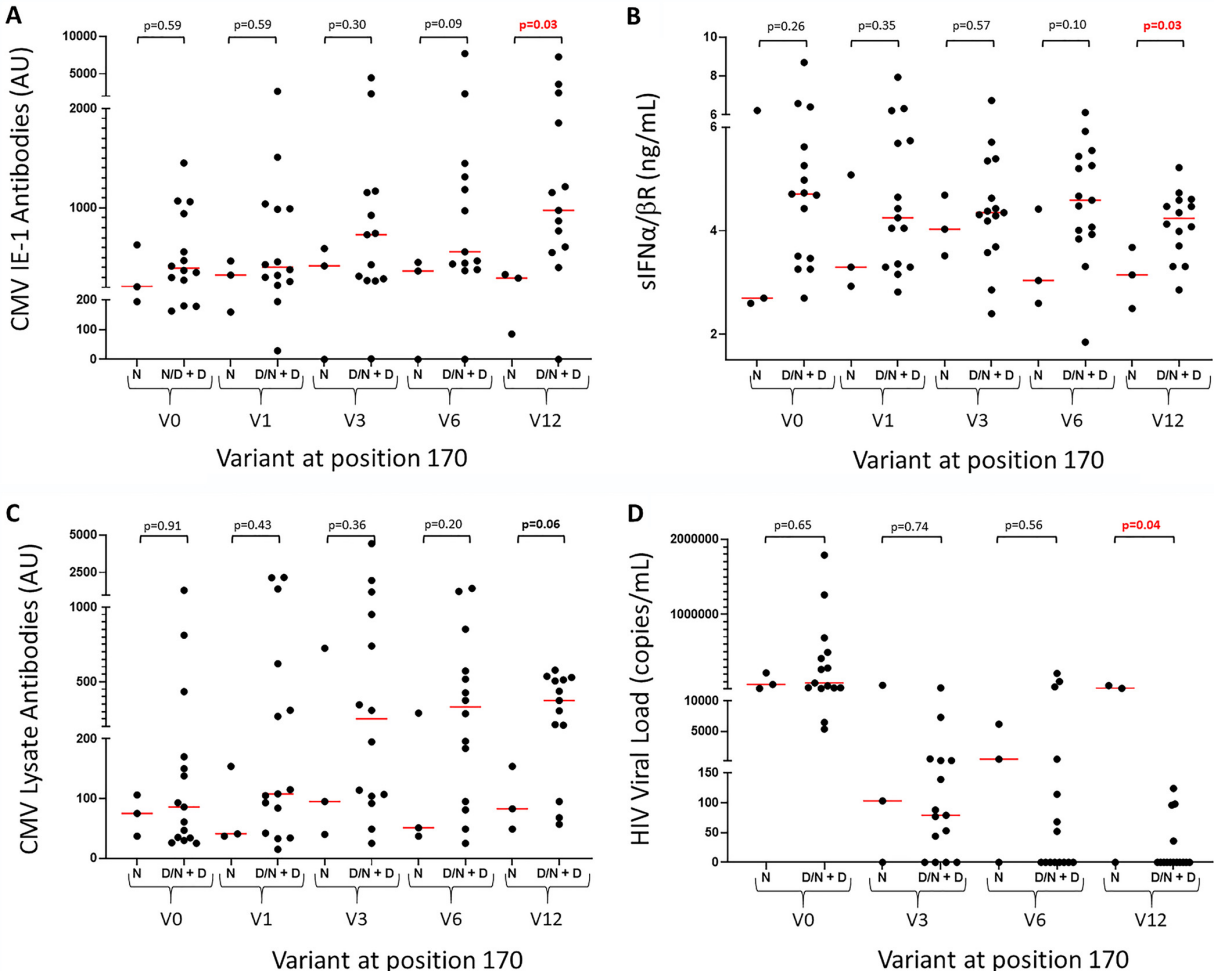
HIV patients carrying HCMV with the US28 N170D variant have higher levels of HCMV-reactive antibodies. (A, C) Comparison of HCMV IE-1 (A) and lysate-reactive antibody (C) levels between HIV patients carrying HCMV with only N at position 170 and those carrying N/D or only D. (B) Comparison of plasma levels of sIFN-α/βR between HIV patients carrying HCMV with only N at position 170 and those carrying N/D or D. (D) Comparison of HIV load in patients carrying HCMV with only N at position 170 and those carrying N/D or D. V0, V1, V3, V6, and V12 represent 0, 1, 3, 6, and 12 months on antiretroviral therapy (ART).

HCMV encoding D at position 170 of US28 was present in 15/18 Indonesian HIV patients participating in the Jakarta, CMV, cardiovascular, antiretroviral, neuropathy, dental, ophthalmology (JakCCANDO) study (20/23 samples), which was sufficient to test statistical associations over the first 12 months on ART. All patients in this cohort were seropositive with a high burden of HCMV attested by very high antibody titers and 52% having HCMV DNA detectable by a simple quantitative PCR (qPCR) before ART. Levels of HCMV-reactive antibodies rose significantly every 3 months for the first year on ART (27). We hypothesize that this reflects a rising capacity to make antibody rather than a rising HCMV burden. Hence, antibody levels at 12 months are probably the best metric of the burden of HCMV. In patients carrying the N170D variant, levels of IE-1-reactive antibodies and soluble type 1 interferon receptor (sIFN-α/βR) were significantly greater after 12 months on ART (Fig. 5A and B; *P* = 0.03 and *P* = 0.03, respectively). The same trend was observed with HCMV lysate antibody (*P* = 0.06) (Fig. 5C). Two out of three patients with only the N variant and 1/20 with mixed infections had detectable HIV RNA (>100 copies/ml) at 12 months (*P* = 0.06, Fisher’s exact test).

## DISCUSSION

Few studies have analyzed HCMV sequences obtained from deep sequencing directly from clinical samples. Suárez et al. utilized high-throughput sequencing of enriched DNA libraries produced directly from clinical samples, which provided insight into HCMV gene recombination (28). However, US28 variants identified in clinical samples have not been linked with predicted ligand binding or clinical outcomes. Here, we use a nested PCR followed by deep sequencing approach on HCMV directly from clinical samples from HIV patients, RTR, healthy controls, and neonates to study variations in US28. The findings carry information regarding regions in which variations are “tolerated.” For example, Caserosa et al. (29) described the significant effects of nonsynonymous mutations at sites 12, 14, and 16 on binding to several chemokines. However, the mutations were created *in vitro* and were not sought in clinical specimens. Only position 15 was affected in virus derived from 60 individuals tested here. The wild-type sequence encodes D (aspartic acid) at this site. While the variant created by Caserosa et al. was an A (alanine), we found only E (glutamic acid), which carries a negative charge similar to D. This suggests that functional variations in this region are not favored by evolution.

An important issue is the prevalence of multivariant infections. While it is plausible that neonates are more likely to be monoinfected, there is little evidence for this in the literature. Here, fewer variations were found in neonatal samples (2 of 4 cases contained multiple variants) than in adult samples. This may reflect recombination events during persistent asymptomatic reactivations or coinfections during childhood or adult life. Of the four neonatal cases, two were asymptomatic, one had hepatitis that resolved spontaneously, and one had sensorineural hearing loss. This infant had a single variant carrying only N170D. Hence, we cannot align any individual variation with clinical outcome in neonates.

Prior evidence of mixed infections is variable, likely reflecting the different populations studied and the sensitivity of the methods used. Sanger sequencing of five HCMV glycoproteins found that 40% of women with primary infections had multiple variants (30), but other studies concluded that multivariant infections are rare (31). This is important clinically as multivariant infections are associated with ganciclovir resistance and graft rejection in organ transplant recipients (32, 33). In mouse models, coinfection with multiple variants can improve the collective viral fitness and consequent growth and dissemination (34).

Haplotype analyses can provide an estimate of the age of the observed mutations, as fixed combinations suggest more ancient mutation events. Here, haplotype US28-1 differed from Toledo only at N170D and was more common in samples from Australia than from Indonesia. The Toledo haplotype was found in a further 10 Australian samples, while samples from Indonesia were more variable. This may reflect the high levels of HCMV replication in the source population, HIV patients beginning ART. Fifty-two percent were HCMV DNA positive when assessed with a simple qPCR detecting UL54 (35). This assay was optimized in our Australian laboratory, but the frequency of detection in Australian RTR was notably lower (13%) (36).

Calculations predicting relative binding energies suggest that many of the variants promote binding of particular chemokines. Most of the variants examined altered amino acids in or near the binding interface with the chemokines or gp120, so their effects on binding are likely to be captured by the modeling procedure used here. While G50C and R267K occur outside this interface, G50C affected binding to CCL4 with limited effects on G protein binding (Table 3; Table S1 in the supplemental material). G50C may reduce US28 flexibility compared to Toledo (reflecting its proximity to the intracellular portion of US28) or US28 activation, stabilizing an active receptor conformation. Arg267 does not come into direct contact with ligands but does contact the receptor N-terminal segment that facilitates ligand binding. Hence, R267K may influence the presentation of the receptor to ligands and indirectly affect ligand binding. Putative effects of G50C and R267K may be corroborated through enhanced sampling simulations investigating receptor activation, which have been routinely used for G protein-coupled receptors (37, 38). Atomic-level simulations have attributed the agonist-independent activity of US28 to an amino acid network evolved to destabilize the receptor’s inactive state (39).

The final section of our paper is a first pass screen linking US28 variants with immune responses to HCMV. While immune pressures may suppress viral replication and reduce *de novo* mutations, the occurrence of several mutations and fixed haplotypes in multiple individuals from different cohorts suggests that the variants were acquired by infection and not generated *de novo* in the individuals described. This argument suggests that variations in US28 alter the footprint of the virus (and not vice versa). Future studies will need to correct for the presence of other mutations in host and viral genes, comorbidities, and sociodemographic factors. This could begin with R267K, as the variant was associated with increased gB-reactive antibody levels in RTR and a healthier FMD (i.e., better endothelial function). Accordingly, a previous study of the same cohort revealed that levels of gB-reactive antibodies were protective of FMD, as assessed in a 3-year follow up (40). Our data suggest that variants carrying R267K may promote this protective response. This is pertinent because gB is under investigation as an HCMV vaccine candidate (41).

The JakCCANDO project provides a longitudinal cohort of HIV patients commencing ART and followed over 12 months, allowing the assessment of the longer-term effects of HCMV variants in a population with a very high CMV burden. CMV DNA sequences were derived from samples collected after 0 to 3 months on ART, and IE-1-reactive antibody levels were elevated in association with the N170D variant at 12 months. The N170D variant was also associated with increased plasma sIFN-α/βR levels. It is plausible that antibody responses to IE-1 and production of interferon-α/β occur early during HCMV reactivation, and, hence, individuals carrying N170D experience more reactivation events. Our modeling suggested that N170D variants may display enhanced binding with CCL13, a chemoattractant for basophils and eosinophils. This could encourage clearance of antibody-coated viral particles, dampening the reactivations. Moreover, N170D may enhance binding to CCL2, which is implicated in the induction of T-cell and monocytic responses that may affect HIV replication. Here, individuals carrying N170D had lower HIV loads after 12 months on ART. While this requires verification in a larger cohort with control for poor adherence to ART, it is interesting that *US28* is expressed during HCMV latency, so its effects are not restricted to periods of active viral replication. US28 is involved in restructuring lipid rafts in host cells mediating cholesterol efflux (42). HIV may utilize lipid rafts to enter or leave a target cell (43, 44).

## CONCLUSION

We have demonstrated diversity in US28 encoded by HCMV carried by HIV patients, RTR, healthy adults, and neonates. The mutations are carried in definable haplotypes, so they may circulate as stable variants. Individual mutations and combinations transmitted in linkage disequilibrium are likely to have differential effects on US28 binding to chemokines and gp120, which may affect the burden of HCMV and/or its clinical footprint in the host. We present preliminary evidence for this in RTR and HIV patients.

## MATERIALS AND METHODS

### RTR and healthy controls from Perth, Western Australia.

Eighty-two RTR were recruited from renal clinics at Royal Perth Hospital (Western Australia). Inclusion criteria were clinical stability greater than 2 years after transplant, no HCMV disease or reactivation within 6 months of sample collection, and no current antiviral treatment. RTR infected with hepatitis B or C were excluded. Ethics approval was obtained from the Royal Perth Hospital Human Research Ethics Committee (approval number EC 2012/155) and endorsed by the Curtin University Human Research Ethics Committee (approval number HRE2021-0044). Participants provided written informed consent (45).

### HIV patients from Jakarta Indonesia.

The JakCCANDO project is a comprehensive survey of clinical and immunological responses to ART undertaken in Cipto Mangunkusumo Hospital’s outpatient clinic (Jakarta, Indonesia) (35). Eighty-two ART-naive HIV patients were enrolled during 2013 to 2014 with <200 CD4 T cells/μl. The study was approved by Universitas Indonesia, Cipto Mangunkusumo Hospital and Curtin University ethics committees. Written consent was obtained from each subject. Examinations were performed before ART initiation (V0) and at months 3, 6, and 12 (V3, V6, and V12). Plasma HIV RNA loads were determined using AmpliPrep/COBAS TaqMan HIV-1 tests (version 2.0), and CD4 T-cell counts were determined using standard flow cytometric techniques (46).

### Australia neonates.

Four deidentified congenital urine samples in virus transport medium were provided by the Department of Microbiology, PathWest Laboratory Medicine, Western Australia. Samples were collected between 1 and 13 days of life and all four had detectable HCMV DNA when assessed by routine hospital assays. Two neonates had symptomatic infections. One had hepatitis attributed to HCMV that spontaneously resolved without antiviral therapy. One had bilateral sensorineural hearing loss and other central nervous system and lymphatic abnormalities and required antiviral therapy.

### Extraction and detection of HCMV DNA.

DNA was extracted from saliva, buffy coat, or urine using FavorPrep blood genomic DNA extraction mini kits (Favorgen, Ping-Tung, Taiwan) and stored at −80°C. HCMV was detected using an in-house qPCR assay with primers targeting the UL54 gene (HCMV DNA polymerase) (36).

### Targeted whole gene amplification.

Primers targeting US28 were designed using Geneious 8.1.9 (https://www.geneious.com) (5′ to 3′: F-AGAAGGGCCAAACACACCAAACG, R-TTCCGGTTCGCTAATCGCACGGA). Reactions were performed in a total volume of 20 μl containing 0.4 μl of MyTaq HS DNA polymerase (Bioline, Meridian Bioscience, Cincinnati, OH), 4 μl of MyTaq reaction buffer, 0.8 μl of 10 μM primers (Sigma-Aldrich, Australia), and 5 μl of DNA diluted 1:2. Cycling conditions were 1 min at 95°C followed by 30 cycles of 15 s at 95°C, 15 s at 58°C, and 1.5 min at 72°C followed by a final extension step of 7 min at 72°C. Amplicons were purified before preparation of DNA libraries using a MO BIO Laboratories Inc. UltraClean PCR clean-up kit (Qiagen, Germany). MyTaq high-sensitivity DNA polymerase is recommended for amplification of products up to 5 kb.

### Preparation of Ion Ampliseq DNA libraries.

Libraries were prepared using an Ion Ampliseq library kit 2.0 with halved reaction volumes and 10 ng of template nucleic acid. The targets were amplified for 30 cycles with an anneal/extension time of 4 min per cycle. During library purification, ethanol was freshly prepared at a 75% concentration. Libraries were quantified using a high-sensitivity DNA kit on a Bioanalyzer 2100 (Agilent, Santa Clara, CA).

### Libraries were sequenced using an Ion Proton sequencer.

Barcoded sample libraries were diluted in low Tris-EDTA (Thermo Fisher Scientific) to reach a final concentration of 100 pmol/liter, and equal volumes of each were pooled. The pooled libraries then underwent template preparation on an Ion Chef system and were loaded onto Ion P1 v3 sequencing chips using an Ion PI Hi-Q Chef kit (Thermo Fisher Scientific). Semiconductor sequencing was performed on an Ion Proton sequencer (Thermo Fisher Scientific) using an Ion PI Hi-Q sequencing kit (Thermo Fisher Scientific) (47).

### Immunological assessments of HCMV.

Plasma stored at −80°C were assessed for HCMV-reactive IgG titers using in-house enzyme-linked immunosorbent assays (ELISAs) based on a lysate of fibroblasts infected with HCMV AD169, recombinant HCMV gB (Chiron Diagnostics, Medfield, MA, USA), or IE-1 protein (Miltenyi Biotech, Cologne, Germany). Results are presented as arbitrary units (AU)/ml based on a standard plasma pool, allowing comparisons between people but not between antigens (27, 45). Soluble receptors for interferon-α/β were assessed in plasma using human IFNAR2 ELISA kits (generously provided by PBL Assay Science, Piscataway, NJ) (35).

### Assessment of vascular pathology.

Ultrasonography was used to assess FMD of the brachial artery after 10 min of rest in Australian RTR and healthy controls (40). FMD assesses the ability of the larger conduit artery to respond to shear stress via endothelial-dependent and endothelial-independent mechanisms.

### Data analysis.

Sequences were mapped to the Toledo reference (GenBank no. GU937742.1) using the tmap tool within the Torrent Suite v 5.10. BAM files mapped to Toledo were loaded into proprietary software, Visual Genomics Analysis Suite (VGAS) (http://www.iiid.com.au/software/vgas) (48). Variants were called if they occurred at a frequency of greater than 10% and had a minimum of 50 in each sample (Table S1). VGAS was also utilized to predict changes in protein sequence.

Amino acid haplotypes and their estimated frequencies were determined using the default parameters of the fastPHASE algorithm with the exception that haplotypes were sampled an additional 5,000 times (49). Haplotypes with a population frequency less than 1% were excluded from analyses. Haplotypes are labeled US28-1 to US28-20 in descending order of their frequencies.

### Statistical analysis.

Continuous data were analyzed with Mann-Whitney nonparametric statistics, and categorical data were analyzed with chi-square or Fisher’s exact tests, as appropriate, using GraphPad Prism version 8 for Windows (GraphPad Software, La Jolla, CA). In individuals where HCMV was sequenced from buffy coat and saliva, only one sample was included in subsequent analyses.

### Modeling of active US28 Toledo bound to chemokines and G proteins.

Prime within Schrodinger Biologics Suite 2018-3 was used for homology modeling and refinement. Chemokine-US28 Toledo-G protein assemblies were prepared using the Heteromultimers facility of Prime, with the chemokine, US28 Toledo, and G protein modeled in separate runs using templates aligned to the relevant portion of the CX3CL1-US28-nanobody crystal structure (PDB 4XT1) (50). The sequence of US28 Toledo was obtained from NCBI (GenBank no. GU937742.1), and US28 Toledo was modeled against US28 in PDB 4XT1. The sequence of Gα_11_, to which US28 is coupled, was obtained from UniProt (accession P29992) (51). The majority of the Gα_11_ structure was modeled against Gα_11_ as contained in the M1 muscarinic acetylcholine receptor-Gα_11_ complex (PDB 6OIJ) (52), while the C-terminal helix contacting the receptor was modeled against Gα_i1_ as contained in the CXCR2-Gα_i1_ complex (PDB 6LFM) (53). To facilitate building of Gα_11_, PDB 6LFM was structurally aligned to PDB 4XT1 by aligning the chemokine receptor components of these structures, following which, Gα_11_ in PDB 6OIJ was aligned to Gα_i1_ in PDB 6LFM. The Gβ_1_ and Gγ_2_ subunits were incorporated directly from PDB 6OIJ. The CX3CL1 structure was used directly from PDB 4XT1. Crystallographic structures of CCL3 (PDB 3FPU) (54), CCL4 (PDB 3TN2) (55), and CCL5 (PDB 5COY) (56) were obtained and aligned to CX3CL1 in PDB 4XT1. To model each chemokine, the majority of the respective crystallographic structures were used, with the N-terminal tail modeled based on CX3CL1 in PDB 4XT1; sequences were aligned using ClustalW within Prime to ensure a reasonable alignment for model building. Following the initial building of the complexes, an implicit membrane was defined on each of these, centered on the seven-transmembrane helical region of US28 Toledo. All residues in the complexes were then subject to energy minimization. In the case of the complexes containing CC-type chemokines, the disulfide bond between the first cysteine residue of the CC motif and the loop between the first and second β-strands was manually introduced prior to energy minimization. All sequence alignments and template selections are illustrated in Tables S3 to S11.

### Modeling of active US28 Toledo bound to representative gp120 proteins and G proteins.

Two gp120 sequences were examined, one from the CRF01_AE strain, which is predominant in Indonesia (GenBank no. MG839510.1), and one from the HXB2 strain, which is predominant in Australia (GenBank no. K03455.1). The structure of the complex of a gp120 bound to CCR5 (PDB 6MEO) (57) was aligned to PDB 4XT1. The desired gp120 sequences were modeled against gp120 in PDB 6MEO. US28 Toledo was modeled against both US28 in PDB 4XT1 and CCR5 in PDB 6MEO in order to achieve a US28 Toledo structure appropriately induced to bind gp120. G protein structures were used as described in the previous section. The gp120-US28 Toledo-G protein assemblies were prepared using the Heteromultimers facility of Prime and refined as described in the previous section.

### Residue scanning calculations.

The residue scanning/affinity maturation tool of Schrodinger Biologics Suite (58) was used to generate variants in US28 Toledo and assess their impact on binding to chemokines and the G protein assembly (i.e., all of the αβγ subunits), adapting our previous work (59). Calculations assessed changes in chemokine binding to the US28-G protein assembly (i.e., chemokine treated as ligand, US28-G protein assembly treated as receptor) and changes in G protein assembly binding to the chemokine-US28 assembly (i.e., αβγ assembly treated as ligand, chemokine-US28 assembly treated as receptor). Use of the previously defined implicit membrane was enabled for all calculations. Variants yielding a predicted change in binding affinity greater in magnitude than 2.0 kcal/mol were considered significantly impacting complex formation, with positive values indicating destabilization and negative values indicating enhanced binding, relative to Toledo.

### Data availability.

Amplicon sequence data have been deposited in NCBI under accession no. SAMN21506830 to SAMN21506889.
