# Supplementary material for: Sequencing Directly from Clinical Specimens Reveals Genetic Variations in HCMV-Encoded Chemokine Receptor US28 That May Influence Antibody Levels and Interactions with Human Chemokines
Source: Microbiol Spectr. 2021 Oct 27;9(2):e00020-21. doi: 10.1128/Spectrum.00020-21 (PMC8549752; doi:10.1128/Spectrum.00020-21)
Supplement: SUPPLEMENTAL FILE 2 — Supplemental material. Download Spectrum.00020-21-s0002.pdf, PDF file, 0.1 MB [file spectrum.00020-21-s0002.pdf]

Supplementary Table 2. Relative binding energies (in kcal/mol) for G protein binding to US28 Toledo variants bound different chemokines and gp120.

Variant	CX3CL1 ΔΔG	CCL2 ΔΔG	CCL3 ΔΔG	CCL4 ΔΔG	CCL5 ΔΔG	CCL13 ΔΔG	gp120 Indonesian ΔΔG	gp120 Australian ΔΔG
D15E	0	0	0	0	0	0	0	0
E18L	0	0	0	0	0	0	0	0
A19D	0	0	0	0	0	0	0	0
T21A	0	0	0	0	0	0	0	0
F25L	0	0	0	0	0	0	-0.01	0
Y40N	-0.07	-0.19	-0.04	-0.16	-0.08	-0.14	-0.03	0
G50C	-0.13	-0.14	-0.02	+7.98	+1.05	+0.03	-0.16	+0.23
N170D	0	0	0	0	0	0	0	0
R267K	0	+0.01	0	0	0	-0.01	0	0

Supplementary Table 3. Sequence alignment for US28 Toledo modelled for chemokine binding

US28 Toledo<br>4XT1_A	1 MTPTTTTAELTTEFDYDEAATPCVFTDVLNQSKPVTLFLYGVVFLFGSIG<br>15 ~~~~~~~~~~~~~~DYDEDATPCVFTDVLNQSKPVTLFLYGVVFLFGSIG<br>**** *******************************
US28 Toledo<br>4XT1_A	51 NFLVIFTITWRRRIQCSGDVYFINLAAADLLFVCTLPLWMQYLLDHNSLA<br>51 NFLVIFTITWRRRIQCSGDVYFINLAAADLLFVCTLPLWMQYLLD~~~~~<br>***
US28 Toledo<br>4XT1_A	101 SVPCTLLTACFYVAMFASLCFITEIALDRYYAIVYMRYRPVKQACLFSIF<br>101 SVPCTLLTACFYVAMFASLCFITEIALDRYYAIVYMRYRPVKQACLFSIF<br>**
US28 Toledo<br>4XT1_A	151 WWIFAVIIAIPHFMVVTKKNNQCMTDYDYLEVSYPIILNVELMLGAFVIP<br>151 WWIFAVIIAIPHFMVVTKKDNQCMTDYDYLEVSYPIILNVELMLGAFVIP<br>*******************:******************************
US28 Toledo<br>4XT1_A	201 LSVISYCYYRISRIVAVSQSRHKGRIVRVLIAVVLVFIIFWLPYHLTLFV<br>201 LSVISYCYYRISRIVAVSQSRHKGRIVRVLIAVVLVFIIFWLPYHLTLFV<br>**
US28 Toledo<br>4XT1_A	251 DTLKLLKWISSSCEFERSLKRALILTESLAFCHCCLNPLLYVFVGTKFRQ<br>251 DTLKLLKWISSSCEFERSLKRALILTESLAFCHCCLNPLLYVFVGTKFRQ<br>**
US28 Toledo<br>4XT1_A	301 ELHCLLAEFRQRLFSRDVSWYHSMSFSRRSSPSRRETSSDTLSDEVCRVS<br>301 ELHCLLAEFR~~~~~~<br>**********
US28 Toledo<br>4XT1_A	361 QIIP<br>~~~~

Supplementary Table 4. Sequence alignment for CCL2.[a]

CCL2	1 MKVSAALLCLLLIAATFIPQGLAQPDAINAPVTCCYNFTNRKISVQRLAS
1DOK_A	23 ~~~~~~~~~~~~~~~~~~~~~~MQPDAINAPVTCCYNFTNRKISVQRLAS
4XT1_B	25 ~~~~~~~~~~~~~~~~~~~~~~XHHGVTKCAI~TCSKMTS~KIPVALLIH
	: * . *: ** ******
CCL2	51 YRRITSSKCPKEAVIFKTIVAKEICADPKQKWVQDSMDHLDKQTQTPKT
1DOK_A	51 YRRITSSKCPKEAVIFKTIVAKEICADPKQKWVQDSMDHLDKQT~~~~~
4XT1_B	51 YQQNQAS~CGKRAIILETRQHRLFCADPKEQWVKDAMQHLDRQ~~~~~~
	**

[a]The regions highlighting are the regions of the respective templates used to model the bound chemokine. Sequence identity/similarity is indicated between the chemokine and the region highlighted. X in the 4XT1_B sequence is pyroglutamic acid.

Supplementary Table 5. Sequence alignment for CCL3.[a]

CCL3	1 MQVSTAALAVLLCTMALCNQFSASLAADTPTAC~~~CFSYTSRQIPQNFI
3FPU_B	24 ~~~~~~~~~~~~~~~~~~~~~~~~SLAADTPTTC~~~CFSYTSRQIPQNFI
4XT1_B	25 ~~~~~~~~~~~~~~~~~~~~~~~~~XHHGVTKCAITCSKMTSK~IPVALL
	* * * *: ******
CCL3	48 ADYFETSSQCSKPGVIFLTKRSRQVCADPSEEWVQKYVSDLELSA
3FPU_B	48 ADYFETSSQCSKPGVIFLTKRSRQVCADPSEEWVQKYVSDLE~~~
4XT1_B	49 IHYQQNQASCGKRAIILETRQHRLFCADPKEQWVKDAMQHLDRQ~
	**

[a]The regions highlighting are the regions of the respective templates used to model the bound chemokine. Sequence identity/similarity is indicated between the chemokine and the region highlighted. X in the 4XT1_B sequence is pyroglutamic acid.

Supplementary Table 6. Sequence alignment for CCL4.[a]

CCL4	1 MKLCVTVLSLLMLVAAFCSPALSAPMGSDPPTACCFSYTARKLPRNFVVD
3TN2_A	24 ~~~~~~~~~~~~~~~~~~~~~~~APMGSDPATACCFSYTARKLPRNFVVD
4XT1_B	25 ~~~~~~~~~~~~~~~~~~~~~~~XHHGVTKCAITCSKMTS~KIPVALLIH
	* * *: *:*******
CCL4	51 YYETSSLCSQPAVVFQTKRSKQVCADPSESWVQEYVYDLELN
3TN2_A	51 YYETSSLCSQPAVVFQTKRSKQVCADPSESWVQEYVYDLEL~
4XT1_B	51 YQQNQASCGKRAIILETRQHRLFCADPKEQWVKDAMQHLDRQ
	**

[a]The regions highlighting are the regions of the respective templates used to model the bound chemokine. Sequence identity/similarity is indicated between the chemokine and the region highlighted. X in the 4XT1_B sequence is pyroglutamic acid.

Supplementary Table 7. Sequence alignment for CCL5.[a]

CCL5<br>5COY_A<br>4XT1_B	1 MKVSAAALAVILIATALCAPASASPYSSDTTPCCFAYIARPLPRAHIKEY<br>27 ~~~~~~~~~~~~~~~~~~~~~~~~~~SSDTTPCCFAYIARPLPRAHIKEY<br>25 ~~~~~~~~~~~~~~~~~~~~~~XHHGVTKCAITCSKMTSK~IPVALLIHY<br>: * :: :********
CCL5<br>5COY_A<br>4XT1_B	51 FYTSGKCSNPAVVFVTRKNRQVCANPEKKWVREYINSLEMS<br>51 FYTSGKCSNPAVVFVTRKNRQVCANPEKKWVREYINSLEMS<br>52 QQNQASCGKRAIILETRQHRLFCADPKEQWVKDAMQHLDRQ<br>***

[a]The regions highlighting are the regions of the respective templates used to model the bound chemokine. Sequence identity/similarity is indicated between the chemokine and the region highlighted. X in the 4XT1_B sequence is pyroglutamic acid.

Supplementary Table 8. Sequence alignment for CCL13.[a]

CCL13<br>2RA4_B<br>4XT1_B	1 MKVSAVLLCLLLMTAAFNPQGLAQPDALNVPSTCCFTFSSKKISLQRLKS<br>26 ~~~~~~~~~~~~~~~~~~~~~~~~~DALNVPSTCCFTFSSKKISLQRLKS<br>25 ~~~~~~~~~~~~~~~~~~~~~~~XHHGVTKCAITCSKMTSK~IPVALLIH<br>: : * :** * ******
CCL13<br>2RA4_B<br>4XT1_B	51 YVITTSRCPQKAVIFRTKLGKEICADPKEKWVQNYMKHLGRKAHTLKT<br>51 YVITTSRCPQKAVIFRTKLGKEICADPKEKWVQNYMKHLG~~~~~~~~<br>51 YQQNQASCGKRAIILETRQHRLFCADPKEQWVKDAMQHLDRQ~~~~~~<br>**

[a]The regions highlighting are the regions of the respective templates used to model the bound chemokine. Sequence identity/similarity is indicated between the chemokine and the region highlighted. X in the 4XT1_B sequence is pyroglutamic acid.

Supplementary Table 9. Sequence alignment for US28 Toledo modelled for gp120 binding.[a]

US28 Toledo	1	MTPTTTTAELTTEFDYDEAATPCVFTDVLNQSKPVTLFLYGVVFLFGSIG
6MEO_B	1	MDYQVSSPIXDINX~~~YTSEPCQKINVKQIAARLLPPLYSLVFIFGFVG
4XT1_A	15	~~~~~~~~~~~~~~DYDEDATPCVFTDVLNQSKPVTLFLYGVVFLFGSIG
		* :: : ** :* : : ** :**:** :*
US28 Toledo	51	NFLVIFTITWRRRIQCSGDVYFINLAAADLLFVCTLPLWMQYLLDHNSLA
6MEO_B	48	NMLVILILINCKRLKSMTDIYLLNLAISDLFFLLTVPFWAHYAAAQWDFG
4XT1_A	51	NFLVIFTITWRRRIQCSGDVYFINLAAADLLFVCTLPLWMQYLLD~~~~~
		***********************************:* * *
US28 Toledo	101	SVPCTLLTACFYVAMFASLCFITEIALDRYYAIVY~~~~MRYRPVKQACL
6MEO_B	98	NTMCQLLTGLYFIGFFSGIFFIILLTIDRYLAVVHAVFALKARTVTFGVV
4XT1_A	101	SVPCTLLTACFYVAMFASLCFITEIALDRYYAIVY~~~~MRYRPVKQACL
		: * *** ::: *:****************** ***********
US28 Toledo	147	FSIFWWIFAVIIAIPHFMVVTKKNN~~~~QCMTDYDYLEVSYPIILN~VE
6MEO_B	148	TSVITWVVAVFASLPGIIFTRSQKEGLHYTCSSHFPYSQYQFWKNFQTLK
4XT1_A	147	FSIFWWIFAVIIAIPHFMVVTKKDN~~~~QCMTDYDYLEVSYPIILN~VE
		************::* : : * : : * : : ::
US28 Toledo	192	LMLGAFVIPLSVISYCYYRISRIVAVSQS~RHKGRIVRVLIAVVLVFIIF
6MEO_B	198	IVILGLVLPLLVMVICYSGILKTLLRCRNEKKRHRAVRLIFTIMIVYFLF
4XT1_A	192	LMLGAFVIPLSVISYCYYRISRIVAVSQS~RHKGRIVRVLIAVVLVFIIF
		::: ************************ *********************
US28 Toledo	241	WLPYHLTLFVDTLKLLKWISSSCEFERSLKRALILTESLAFCHCCLNPLL
6MEO_B	248	WAPYNIVLLLNTFQEFFGLNN~CSSSNRLDQAMQVTETLGMTHCCINPII
4XT1_A	241	WLPYHLTLFVDTLKLLKWISSSCEFERSLKRALILTESLAFCHCCLNPLL
		**
US28 Toledo	291	YVFVGTKFRQELHCLLAEFRQRLFSRDVSWYHSMSFSRRSSPSRRETSSD
6MEO_B	297	YAFVGEKFRNYLLVFFQ~~~~~~~~~~~~~~~~~~~~~~~~~~~~~~~~~
4XT1_A	291	YVFVGTKFRQELHCLLAEFR~~~~~~~~~~~~~~~~~~~~~~~~~~~~~~
		********************
US28 Toledo	341	TLSDEVCRVSQIIP
6MEO_B		~~~~~~~~~~~~~~
4XT1_A		~~~~~~~~~~~~~~

[a]The regions highlighting are the regions of the respective templates used to model the gp120-bound conformation of US28 Toledo. Sequence identity/similarity is indicated between the receptor and the region highlighted. X in the 6MEO_B sequence indicates an unknown residue.

Supplementary Table 10. Sequence alignment for gp120 Indonesian.

gp120_ID<br>6MEO_G	1<br>29	MRVKKTQMNWLSWWKWGTLILGLVIMCNASDNLWVTVYYGVPVWKDAETT<br>~~~~~~~~~~~~~~~~~~~~~~~~~~~~~~DNLWVTVYYGVPVWKEATTT<br>***************:* **
gp120_ID<br>6MEO_G	51<br>49	LFCASDAKAHETEVHNVWATHACVPTDPNPQELPLKNVTENFNMWKNPMA<br>LFCASDAKAYKAEVHNVWATHACVPTDPNPQEIVLENVTENFNMWKNNMV<br>*********:: ********************: *:*********** *
gp120_ID<br>6MEO_G	101<br>99	EQMHEDVISLWDQSLKPCVKLTPLCVTLNCTNAKLTNVTDVSNTTESNPT<br>EQMHEDIISLWDQSLKPCVKLTPLCVTLNC~~~~~~~~~~~~~~~~~~~~<br>******:***********************
gp120_ID<br>6MEO_G	151	ESTTRNNTIETDEVKNCTFNVTTELTDMTKQVHALFYKLDIVQINDRSVN<br>~~~~~~
gp120_ID<br>6MEO_G	201<br>184	NNSSSGKYMLINCNTSVIKQACPKISFDPIPIHYCAPAGYAILKCKDKKF<br>IDNTS~~YRLTSCNTSVITQACPKVTFEPIPIHYCTPAGYAILKCNGKKF<br>:::* * * :****** *****::*:******* ********* ***
gp120_ID<br>6MEO_G	251<br>232	NGIGPCNNVSSVQCTHGIRPVVSTQLLLNGSLAEEEIIIRSENITNNAKN<br>NGTGPCTNVSTVQCTHGIKPVVSTQLLLNGSLAEEDIVIRSENLTNNAKT<br>** *** ***:*******:****************:*:*****:*****
gp120_ID<br>6MEO_G	301<br>282	IIVHLNKSIEISCIRAYKNTRTSTHMVPGRTRFRTGGIIGDIKKAHCEIN<br>IIVQLKDPVDINCTRPNNNTRKSIHIGPGRAFYATGDIIGDIRQAHCNLS<br>*** * ::*:* * *** * *: *** : ** *****::*** ::
gp120_ID<br>6MEO_G	351<br>332	GTTWNETLEQVKRKLEKYFPNKTIIFQPQPHAGGDPEITMHHFNCRGEFF<br>RAQWNDTLSKIVTKLREQFENKTIKFQP~~PSGGDPEIVFHSFNCGGEFF<br>**:** :: ** : * **** *** :****** * *** ****
gp120_ID<br>6MEO_G	401<br>380	YCNTTDLFNNNRTDGT~~~VKLPCRIKQFVNMWQRVGQAIYAPPISGTLN<br>YCNTTQLFNSTWTNNTEDTITLPCRIKQIVNMWQEVGKAMYAPPIKGKIK<br>***** ***: *: * : ******* ***** **:*:***** * :
gp120_ID<br>6MEO_G	448<br>430	CTSNITGIILTRDGADTNSTRNETSSSNETEIFRPIGGDMRDNWRSELYK<br>CSSNITGLLLTRDGGNNE~~~~~~~~MNTTEIFRPGGGDMRDNWRSELYK<br>*:*****::***** : * ****** **************
gp120_ID<br>6MEO_G	498<br>480	YKVVQIEPLGIAPTRAKR<br>YKVVRIEPLG~~~~~~~~<br>****:*****

Supplementary Table 11. Sequence alignment for gp120 Australian.

gp120_AU<br>6MEO_G	1<br>29	MRVKEKYQHLWRWGWRWGTMLLGMLMICSATEKLWVTVYYGVPVWKEATT<br>~~~~~~~~~~~~~~~~~~~~~~~~~~~~~~~DNLWVTVYYGVPVWKEATT<br>: *****************
gp120_AU<br>6MEO_G	51<br>49	TLFCASDAKAYDTEVHNVWATHACVPTDPNPQEVVLVNVTENFNMWKNDM<br>TLFCASDAKAYKAEVHNVWATHACVPTDPNPQEIVLENVTENFNMWKNNM<br>*********** ********************:** ***********:*
gp120_AU<br>6MEO_G	101<br>99	VEQMHEDIISLWDQSLKPCVKLTPLCVSLKCTDLKNDTNTNSSSGRMIME<br>VEQMHEDIISLWDQSLKPCVKLTPLCVTLNCIDN~~~~~~~~~~~~~~~~<br>***************************:* * *
gp120_AU<br>6MEO_G	151<br>187	KGEIKNCSFNISTSIRGKVQKEYAFFYKLDIIPIDNDTTSYKLTSCNTSV<br>~~~~~~~~~~~~~~~~~~~~~~~~~~~~~~~~~~~~~~TSYRLTSCNTSV<br>***:********
gp120_AU<br>6MEO_G	201<br>199	ITQACPKVSFEPIPIHYCAPAGFAILKCNNKTFNGTGPCTNVSTVQCTHG<br>ITQACPKVTFEPIPIHYCTPAGYAILKCNGKKFNGTGPCTNVSTVQCTHG<br>********:********* ***:****** * ******************
gp120_AU<br>6MEO_G	251<br>249	IRPVVSTQLLLNGSLAEEEVVIRSVNFTDNAKTIIVQLNTSVEINCTRPN<br>IKPVVSTQLLLNGSLAEEDIVIRSENLTNNAKTIIVQLKDPVDINCTRPN<br>*:****************::**** * *:********* *:*******
gp120_AU<br>6MEO_G	301<br>299	NNTRKRIRIQRGPGRAFVTIG~KIGNMRQAHCNISRAKWNNTLKQIASKL<br>NNTRKSIHIGPGR~~AFYATGDIIGDIRQAHCNLSRAQWNDTLSKIVTKL<br>***** * * * ** * **::******:***:**:** :* :**
gp120_AU<br>6MEO_G	350<br>347	REQFGNNKTIIFKQSSGGDPEIVTHSFNCGGEFFYCNSTQLFNSTWFNST<br>REQF~ENKTIKFQPPSGGDPEIVFHSFNCGGEFFYCNTTQLFNSTWTNNT<br>**** **** *: ******** *************:******** *:*
gp120_AU<br>6MEO_G	400<br>396	WSTEGSNNTEGSDTITLPCRIKQIINMWQKVGKAMYAPPISGQIRCSSNI<br>~~~~~~~~~~~EDTITLPCRIKQIVNMWQEVGKAMYAPPIKGKIKCSSNI<br>************:****:********** *:*:*****
gp120_AU<br>6MEO_G	450<br>435	TGLLLTRDGGNSN~NESEIFRPGGGDMRDNWRSELYKYKVVKIEPLGVAP<br>TGLLLTRDGGNNEMNTTEIFRPGGGDMRDNWRSELYKYKVVRIEPLG~~~<br>***********: * :************************:*****
gp120_AU<br>6MEO_G	499	TKAKR<br>~~~~~
